# Genomic signature and evolutionary history of completely cleistogamous lineages in the non-photosynthetic orchid *Gastrodia*

**DOI:** 10.1098/rspb.2025.0574

**Published:** 2025-05-21

**Authors:** Kenji Suetsugu, Shun K. Hirota, Takashi Makino, Yoshihisa Suyama, Shingo Kaneko, Kenji Fukushima

**Affiliations:** ^1^ Department of Biology, Graduate School of Science, Kobe University, Kobe, Hyogo, Japan; ^2^ Institute for Advanced Research, Kobe University, Kobe, Hyogo, Japan; ^3^ Botanical Gardens, Osaka Metropolitan University, Katano, Osaka, Japan; ^4^ Graduate School of Life Sciences, Tohoku University, Miyagi, Japan; ^5^ Graduate School of Agricultural Science, Tohoku University, Miyagi, Japan; ^6^ Faculty of Symbiotic Systems Science, Fukushima University, Fukushima, Japan; ^7^ National Institute of Genetics, Mishima, Shizuoka, Japan; ^8^ Graduate Institute for Advanced Studies, SOKENDAI, Mishima, Shizuoka, Japan

**Keywords:** complete cleistogamy, deleterious mutations, genomic selfing syndrome, speciation, mycoheterotrophy

## Abstract

Despite a long-standing interest since Darwin’s time, the genomic implications of obligate self‐fertilization remain elusive. Complete cleistogamy—the obligate production of closed, self‐pollinating flowers—represents an extreme reproductive strategy. Here, we present the genomic profiles and evolutionary history of two lineages of the mycoheterotrophic orchid *Gastrodia*, both of which independently acquired complete cleistogamy, based on detailed sampling and a combination of simple sequence repeat (SSR), multiplexed ISSR genotyping by sequencing (MIG-seq) and RNA‐seq data. Our analysis reveals clear species delimitation, with no evidence of introgression between the completely cleistogamous species and their co‐occurring allogamous sisters. Intriguingly, all analyses indicate that both the completely cleistogamous *Gastrodia* species and their allogamous sisters exhibit genetic profiles typical of self‐pollinating plants. This pattern suggests that their ancestors, probably bearing allogamous flowers, had already evolved mechanisms to mitigate the deleterious effects of selfing, potentially facilitating the emergence of complete cleistogamy through benefits such as reproductive assurance, enhanced colonization ability and species reinforcement. Meanwhile, further analyses suggest that complete cleistogamy evolved very recently (possibly within the last 1000–2000 years) in these two *Gastrodia* lineages. Combined with the scant evidence of complete cleistogamy outside *Gastrodia*, our findings imply a limited and ephemeral role for complete cleistogamy in plant speciation.

## 1. Introduction

Charles Darwin once remarked, ‘It is hardly an exaggeration to say that Nature tells us, in the most emphatic manner, that she abhors perpetual self-fertilisation’ [1, p. 359]. Yet, self-fertilization (selfing) has evolved repeatedly across the angiosperms, often providing crucial reproductive assurance in environments where pollinators or conspecific mates are scarce [2,3]. Despite these apparent benefits, prolonged reliance on selfing can exacerbate genetic drift, promote the accumulation of deleterious mutations and reduce the effective population size (*N_e_*) [4,5]. This duality, where selfing emerges frequently yet carries inherent genetic risks, remains a central enigma in plant evolutionary biology [3,6].

Many studies on the evolution of self-fertilization highlight its immediate advantages: selfers can reproduce even in the absence of pollinators or within sparsely distributed populations [3,7–9]. Meanwhile, transitions to selfing are often accompanied by a genomic ‘selfing syndrome’, characterized by reduced heterozygosity and elevated burdens of deleterious alleles [6,10–13]. Notably, predominantly selfing species such as *Arabidopsis thaliana* and *Capsella rubella* still permit occasional outcrossing, which introduces heterozygosity and may buffer against genomic decay [14–17]. Consequently, it remains unclear how lineages that are entirely isolated from outcrossing (i.e. obligate selfers) surmount these genetic constraints and over what timescale obligate selfing can persist as an evolutionarily stable strategy [9].

Although autonomous selfing probably evolved primarily to ensure reproductive assurance, it may also function as an effective barrier to heterospecific mating, thereby enhancing reproductive isolation [3,17–19]. While a single reproductive barrier is often insufficient to prevent interspecific hybridization [20,21], complete cleistogamy—the obligate formation of closed, self-pollinating flowers—constitutes an extreme form of reproductive isolation, probably eliminating gene flow with closely related taxa [22,23]. Therefore, complete cleistogamy may be selected not only in response to colonization challenges but also as a reinforcement mechanism during speciation, provided that the deleterious effects of inbreeding are mitigated. However, owing to the extreme rarity of complete cleistogamy [9,24], such possibilities have remained largely unexplored.

The mycoheterotrophic orchid genus *Gastrodia* offers an exceptional system for examining the dynamics of obligate selfing and complete cleistogamy, as it includes several species that produce exclusively cleistogamous flowers, with no apparent potential for outcrossing [25–29]. While cleistogamy has independently evolved across diverse lineages of angiosperms, most cleistogamous species also produce open, chasmogamous flowers [9,24]. To date, *Gastrodia* appears to be the only genus comprising multiple species that exhibit complete cleistogamy [22]. Unlike commonly studied models of selfing, such as *Mimulus nasutus* and *C. rubella*, which retain some capacity for outcrossing [12,19], the completely cleistogamous species of *Gastrodia* (e.g. *Gastrodia takeshimensis* and *Gastrodia kuroshimensis*) probably rely entirely on obligate self-fertilization [22,23]. Intriguingly, these lineages are typically restricted to islands where closely related chasmogamous and allogamous *Gastrodia* species are also present. Thus, beyond the classical rationales for selfing, such as providing reproductive assurance and facilitating colonization of unoccupied habitats [7–9], this distributional pattern suggests that obligate selfing may function as a mechanism of reinforcement, effectively preventing interspecific gene flow and maintaining species integrity [30].

To elucidate the origins and timing of obligate selfing and to determine whether these species exhibit any genomic signals of introgression, we employed an integrative genomic framework. First, we used hypervariable simple sequence repeat (SSR) markers, which evolve rapidly through *de novo* mutations and effectively capture recent demographic events [31,32]. Second, we applied multiplexed ISSR genotyping by sequencing (MIG-seq), a reduced-representation genomic technique well-suited for detecting single-nucleotide polymorphisms (SNPs) [33], providing robust insights into historical genome-wide variation even in non-model organisms [34,35]. Finally, we analysed low-copy transcript data from RNA-seq to characterize heterozygous mutations and the accumulation of deleterious variants [36], enabling evaluation of typical features associated with the selfing syndrome in *Gastrodia*. By integrating these complementary approaches, we characterized genomic traits linked to obligate selfing, focusing on two probably independently evolved cleistogamous species, *G. kuroshimensis* and *G. takeshimensis*, in comparison with their respective allogamous relatives, *G. foetida* and *G. fontinalis* [23].

Our study addresses three key questions: (i) how recently did *G. takeshimensis* and *G. kuroshimensis* diverge from their allogamous relatives, and can we estimate the timing of the evolution of complete cleistogamy?; (ii) is there evidence of introgression between cleistogamous and chasmogamous species in sympatric regions?; and (iii) under what conditions does *Gastrodia* probably favour complete cleistogamy? For instance, are chasmogamous congeners already partially inbred, thereby predisposing them to a shift toward obligate selfing? Through genomic analyses, this study offers novel insights into the evolutionary trajectory of complete cleistogamy, shedding light on plant speciation, mating system transitions and the long-term sustainability of obligate selfing.

## 2. Material and methods

### (a) Study species

**Figure 1.**
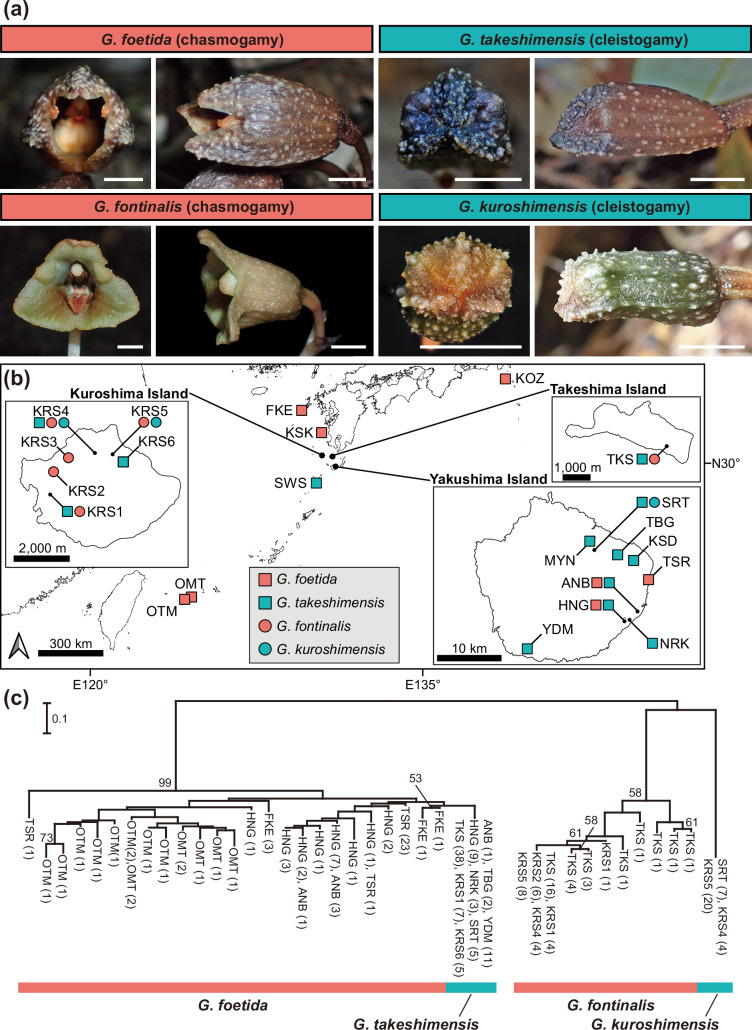
Four *Gastrodia* species were investigated, and their genomic profiles were based on SSR genotype data. (a) Flower morphology (front and lateral views). Scale bar: 5 mm. (b) Map showing the sampling localities. (c) Unrooted neighbour-joining phylogenetic tree based on *D*_A_ distances [39] calculated from SSR genotypes. Two well-supported clades were recovered: one comprising *G. foetida* and *G. takeshimensis* and the other comprising *G. fontinalis* and *G. kuroshimensis*, each with 99% bootstrap support. Numbers in parentheses denote the number of individuals sharing an identical multilocus genotype. Nodes with bootstrap support below 50% are not shown.

*Gastrodia* species are fully mycoheterotrophic orchids that depend on saprotrophic fungi [37,38]. Their flowers, like those of many orchids, are zygomorphic, comprising an outer whorl of three sepals and an inner whorl of three petals, including a specialized labellum. In all *Gastrodia* species, the sepals and petals other than the labellum are partially fused, forming a five-lobed perianth tube. In the chasmogamous species *G. foetida* and *G. fontinalis*, the dehiscence zone at the free part of the sepals consistently opens, whereas, in the cleistogamous sister species *G. takeshimensis* and *G. kuroshimensis*, it remains fused throughout the reproductive period (figure 1a). Consequently, the mature flowers of the cleistogamous species retain a more juvenile, bud-like appearance [23]. In *G. foetida* and *G. fontinalis*, a pronounced rostellum prevents autonomous self-pollination, requiring pollination by drosophilid flies for fruit set [40]. By contrast, the absence of a well-developed rostellum in *G. takeshimensis* and *G. kuroshimensis* enables autonomous self-pollination [26,28].

Although *G. foetida* was long considered synonymous with *Gastrodia nipponica*, a recent taxonomic study has shown that *G. foetida* is morphologically and phylogenetically distinct from *G. nipponica* [41]. These species also exhibit geographical segregation, with *G. nipponica* occurring north of the Kyushu mainisland and *G. foetida* inhabiting the Ryukyu Islands and possibly Taiwan [41]. While our previous work on the development of completely cleistogamous flowers used the name *G. nipponica* as the sister species of *G. takeshimensis* [23]*, G. nipponica sensu* [23] is now recognized as *G. foetida*.

The distributions of *G. takeshimensis* and *G. kuroshimensis*, both endemic to the northern Ryukyu, generally overlap with those of their allogamous sister species [26,28,42,43]. Although the cleistogamous taxa and their allogamous sisters occasionally occur in close proximity, no individuals exhibiting intermediate morphological traits indicative of hybridization have been observed even in sympatric sites [26,28]. Given that these four *Gastrodia* species can reproduce clonally through tuber production, with clonal propagation documented up to approximately 60 cm [44,45], individuals located at least 1 m apart were collected to minimize the possibility of sampling multiple clonal ramets. Consequently, 91 individuals of *G. foetida*, 119 of *G. takeshimensis*, 81 of *G. fontinalis* and 41 of *G. kuroshimensis* were sampled, covering nearly all known populations (and all existing populations in the cleistogamous species) (figure 1b; electronic supplementary material, table S1). Previous morphological and phylogenetic evidence strongly supports the sister-species relationships between *G. fontinalis–G. kuroshimensis* and *G. foetida–G. takeshimensis* [23,26,28,41,46]. To further substantiate these relationships, we conducted an expanded MIG-seq-based phylogenetic analysis incorporating additional morphologically similar species (see the electronic supplementary material, note S1).

### (b) Simple sequence repeat marker analysis

Genomic DNA was extracted from fresh or silica‐dried samples using the DNeasy Plant Mini Kit (Qiagen, Venlo, Netherlands). A total of 18 SSR markers previously developed (*Gtake009, Gtake011, Gtake012, Gtake015, Gtake020, Gtake021, Gtake022, Gtake029, Gtake032, Gtake037, Gtake042, Gtake043, Gfont027, Gfont028, Gfont034, Gfont035, Gfont043* and *Gfont048*; [43,46]) were used to genotype the four *Gastrodia* species (electronic supplementary material, table S1). Polymerase chain reaction (PCR) amplification was performed in 5.0 μl reaction volumes using the QIAGEN Multiplex PCR Kit. Each reaction comprised 10 ng of genomic DNA, 2.5 μl of Multiplex PCR Master Mix and 0.2 μM each of a fluorescently labelled forward primer and a reverse primer. The amplification protocol included an initial denaturation at 95°C for 15 min, followed by 33 cycles at 94°C for 30 s, 57°C for 1.5 min, and 72°C for 1 min, with a final extension at 60°C for 30 min. An ABI PRISM 3130 Genetic Analyzer (Applied Biosystems, MA, USA) and GeneMarker software (Softgenetics, PA, USA) were used to determine product sizes.

Only samples that successfully amplified all 18 loci (68 *G. foetida*, 82 *G*. *takeshimensis*, 54 *G*. *fontinalis* and 31 *G*. *kuroshimensis* individuals) were used for subsequent analyses. Genetic variation within each population was assessed by calculating the average number of alleles per locus (*A*), allelic richness (*R*_S_), observed heterozygosity (*H*_O_) and expected heterozygosity (*H*_E_) using GenAlEx v. 6.5 [47], with allelic richness computed using FSTAT v. 2.9.3 [48]. Genetic relationships among multilocus genotypes were assessed by constructing an unrooted neighbour-joining tree based on Nei’s genetic distance *D*_A_ [39], using Populations v. 1.2.31 [49].

To estimate the number of generations over which fixed alleles at SSR markers could be maintained despite their high mutation rate, we used the mutation rate directly estimated in *A. thaliana* and considered three scenarios for effective population sizes. In an obligate self-fertilizing lineage, the probability (*P_t_*) that no new alleles arise through mutation at all *l* SSR loci—with a haploid mutation rate *μ*—after *t* generations is given by:


$$P_{t}=(1-\mu {)}^{\left(l\cdot t\right)}.$$

Each line is expected to produce new alleles with a 2*µ* probability per locus per generation, but only half of these new alleles will be fixed in the subsequent generations of selfed propagation. Furthermore, within a population with an effective population size *N*_e_, the probability *P_i_* that only an identical genotype is observed across all lines can be expressed as:


$$P_{i}=(P_{t}{)}^{N_{e}}.$$

The mutation rate of SSR markers was estimated by genotyping *A. thaliana* mutation accumulation lines targeting dinucleotide repeat loci [50]. Because the mutation rate varies with dinucleotide motif types and the number of repeats (ranging from 2.0 × 10⁻³ to 5.0 × 10⁻⁵) [50], we used the lowest rate (4.96 × 10⁻⁵) to conservatively estimate the number of generations during which the same genotype is maintained. In our study, the number of loci (*l*) considered is six (those with dinucleotide repeats among the SSR markers). Based on current population sizes (approx. 50 individuals for *G. kuroshimensis*, 250 for *G. fontinalis* and *G. foetida* and 500−1000 for *G. takeshimensis*), *N*ₑ was evaluated for three scenarios (50, 100 and 500) to estimate the probability that a new allele will not become fixed in the population.

### (c) Multiplexed ISSR genotyping by sequencing analysis

Genomic DNA was extracted from silica‐dried samples using the cetyltrimethylammonium bromide method [51]. The MIG‐seq library was prepared following Suyama *et al*. [33]. Paired‐end sequencing (2 × 80 bases) was performed on an Illumina MiSeq sequencer using a MiSeq Reagent Kit v. 3 (150 cycles) (Illumina, San Diego, CA, USA). The raw sequencing data have been deposited in the DDBJ Sequence Read Archive under accession number PRJDB18852. As with the SSR data, only samples yielding robust amplification and reliable SNP calls were used; consequently, 78 individuals of *G. foetida*, 107 of *G. takeshimensis*, 60 of *G. fontinalis* and 34 of *G. kuroshimensis* were used for subsequent analyses (electronic supplementary material, table S1).

The primer sequences and low-quality reads were removed using Trimmomatic v. 0.39 [52]. The quality‐controlled reads were aligned to the genome of *Gastrodia menghaiensis*, which is the most closely related species with an available genome [53], using BWA v. 0.7.17 [54] with default parameters. Although the *G. menghaiensis* genome was used as a reference for mapping MIG‐seq reads from our focal species, *G. menghaiensis* itself was not included in the analysis owing to the unavailability of MIG‐seq data.

SNP calling was performed using the ‘gstacks’ and ‘populations’ modules of the Stacks pipeline v. 2.65 [55]. Stacks use the alpha value in gstacks as a measure of evidence in genotyping instead of read depth [56]. In this study, both alpha values—var‐alpha and gt‐alpha—were set to 0.001 (reduced from their default values of 0.01 and 0.05, respectively). Using the Populations program, SNPs with high heterozygosity (*H*_O_ ≥ 0.6) were removed, because excess heterozygosity may result from artefactual loci arising from multiple paralogous genomic regions [57]. Additionally, SNPs with a minor allele detected in fewer than three samples throughout the entire dataset were excluded.

SNPs were further selected using minimum proportion thresholds of 0.1 and 0.7 for samples retaining each SNP (the Populations parameter, *R*, was set to 0.1 and 0.7, respectively). Based on SNP data with a threshold of *R* = 0.1, the maximum likelihood phylogeny was reconstructed with RAxML v. 8.2.10 [58], employing a GTR substitution model with Lewis’ ascertainment bias correction and 1000 bootstrap iterations. A neighbour-net network was generated using SplitsTree v. 4.14 [59], based on an uncorrected *p*-distance matrix while excluding ambiguous sites. In addition to SNP detection across all four species, we conducted SNP detection hierarchically within sister species [60] to achieve higher resolution using lineage-specific SNPs (electronic supplementary material, figure S2). With a threshold of *R* = 0.7, subsequent analyses were conducted separately for three datasets: all four species and the sister pairs (*G. foetida* and *G. takeshimensis* and *G. fontinalis* and *G. kuroshimensis*). For this ‘SNP dataset for species pairwise comparisons’, only the first SNP from each locus was considered to avoid linked SNPs. The population structure for both pairs was analysed using Structure v. 2.3.4 [61] under the admixed and correlated allele frequency model to examine the extent of admixture between chasmogamous and cleistogamous species. The number of clusters (*K*) was set from 1 to 10 based on 30 independent runs for each *K* and 100 000 Markov chain Monte Carlo (MCMC) steps after 100 000 burn-in steps. Log-likelihoods for each cluster were calculated, and optimal *K* values were identified using the Delta *K* method [62] via Structure Harvester v. 0.7 [63], with outcomes visualized using Clumpak v. 1.1 [64]. It should be noted that, because our analyses were performed separately by sister species pairs, we could not directly assess potential introgression between the cleistogamous lineages. Consequently, one might be concerned that such introgression could account for the emergence of cleistogamy in *G. kuroshimensis* and *G. takeshimensis*. However, this possibility was previously ruled out using the ABBA-BABA test with *D* statistics based on RNA-seq data [23]. Therefore, genetic convergence, where independent mutations result in similar phenotypes, is the most likely explanation for the independent evolution of cleistogamy, making the separate analysis of species pairs appropriate.

Population statistics, including the number of private alleles, observed heterozygosity (*H*_O_), expected heterozygosity (*H*_E_), the population’s average inbreeding coefficient (*F*_IS_) and nucleotide diversity (*π*), were calculated using the SNP dataset for species pairwise comparisons with the Populations program in Stacks. To visualize shared alleles between sister species, histograms of ancestral allele frequencies were constructed, considering the allele predominant in the chasmogamous species as ancestral.

Finally, we estimated the demographic history of the sister pairs (*G. foetida–G. takeshimensis* and *G. fontinalis–G. kuroshimensis*), including the number of generations since divergence, using the approximate Bayesian computation (ABC) algorithm implemented in DIYABC v. 2.1.0 [65]. Given that (i) complete cleistogamy leads to strong reproductive isolation, and (ii) our molecular analyses detected no signs of introgression, we adopted a simple divergence model that assumes no hybridization occurred after speciation.

### (d) Analysis of heterozygous single-nucleotide variants in low-copy messenger RNA sequences

*Gastrodia foetida* and *G. takeshimensis* individuals (*n* = 9 each) were collected on Yakushima Island, Kagoshima Prefecture, Japan, in early to mid-April 2017. Concurrently, individuals of *G. fontinalis* and *G. kuroshimensis* (*n* = 9 each) were obtained from Kuroshima Island, Kagoshima Prefecture, Japan, during mid- to late-April 2017 (electronic supplementary material, table S1). Owing to the higher cost per RNA‐seq library and the stringent preservation requirements for sampling on remote islands, fewer samples were analysed for RNA‐seq than for SSR or MIG‐seq. Samples were minced into fragments of less than 3 mm in length and immediately submerged in RNAlater stabilization solution (Sigma-Aldrich, St Louis, MO, USA). They were stored in a cool box during transport to the laboratory and subsequently preserved at −80°C until RNA extraction.

Total RNA was prepared following Suetsugu *et al*. [41]. For library preparation, only samples with RNA integrity numbers of 8.0 or higher were selected. Libraries were constructed using the MGI Easy RNA Directional Library Prep Set (MGI, Shenzhen, China), and paired-end sequencing (2 × 150 bases) was performed on a DNBSEQ-G400RS instrument (MGI). The RNA‐seq data accession numbers are PRJDB10993 for *G. foetida*, PRJDB10965 for *G. takeshimensis*, PRJDB10966 for *G. fontinalis* and PRJDB10992 for *G. kuroshimensis*.

To accurately detect heterozygous loci within individuals, each RNA‐seq sample was assembled and analysed separately. *De novo* transcriptome assembly was performed using Trinity v. 2.11.0 [66] after pre-processing reads with fastp v. 0.20.1 [67]. Open reading frames were predicted using TransDecoder v. 5.5.0 (https://github.com/TransDecoder/TransDecoder). To focus on primary transcripts of well-conserved genes, we extracted splicing isoforms with the longest coding sequences using CDSKIT v. 0.9.2 (https://github.com/kfuku52/cdskit) and selected low-copy genes via BUSCO v. 5.3.2 [68] with the embryophyta_odb10 dataset. Primary transcripts identified as single-copy, duplicated or fragmented were included in downstream analyses.

To identify heterozygous single-nucleotide variants (SNVs), RNA‐seq reads were mapped to the low-copy reference transcript set using BWA v. 0.7.13 [69], and SNVs were called using SAMtools v. 1.3 [70]. Heterozygous SNVs, indicative of genetic diversity, were quantified per kilobase (kb) of total transcript length. The impact of non-synonymous SNVs on protein function was assessed using PROVEAN v. 1.1.5 with the NCBI NR database (downloaded 13 October 2013), as described previously [36,71]. Typically, a PROVEAN score of −2.5 or lower predicts deleterious variation, whereas scores above −2.5 are considered neutral. In this study, non-synonymous variations with an absolute PROVEAN score greater than 2.5 were classified as deleterious, given the difficulty in differentiating derived from ancestral variants at heterozygous loci. We then calculated the ratio of deleterious amino acid variations among heterozygous SNVs and identified transcripts harbouring non-synonymous SNVs. These SNV-based metrics were statistically compared between sister species pairs using a Student’s *t*‐test.

To compare heterozygous SNV metrics between *Gastrodia* and other self-pollinating lineages, we retrieved angiosperm RNA‐seq data from GenBank using AMALGKIT v. 0.6.5.6 (https://github.com/kfuku52/amalgkit) and applied the same analyses to individual samples. These database-derived samples included model lineages for studying genomic selfing syndrome, such as *Capsella* [10,11,17,72,73]. Because mycoheterotrophy may represent an adaptation to low-light environments that hinders pollinator attraction (potentially increasing selfing rates) [22,74,75], we included other mycoheterotrophic plants as controls. To avoid biases related to sequencing data quality, only paired-end RNA‐seq datasets with transcriptome assemblies achieving BUSCO completeness scores above 50% were used. After screening, the methodologies described above were applied to obtain SNV-based metrics across 301 RNA-seq samples from 37 angiosperm species, including 33 samples from four *Gastrodia* species sequenced in this study (electronic supplementary material, table S2).

### (e) Phylogenetic generalized least squares

For each species, one sample from the low-copy transcript set with the highest BUSCO completeness was selected for species tree inference. This inference was performed using the maximum-likelihood method in IQ-Tree v. 2.2.5 [76] with the LG+R model. The concatenated multiple alignments of amino acid sequences, encompassing a total of 790 412 sites, were generated using MAFFT v. 7.508 [77] and trimmed with trimAl v. 1.4.1 [78], treating duplicated and missing genes as missing data [79]. Divergence times were estimated using MCMCtree in the PAML package v. 4.10.7 [80]. Time constraints were prepared using data from timetree.org and processed with NWKIT v. 0.14.2 using the ‘--timetree ci’ option (https://github.com/kfuku52/nwkit). We conducted a phylogenetic generalized least squares (PGLS) analysis using Rphylopars v. 0.3.9 [81] to detect associations between SNV-based metrics and the selfing syndrome across species. The selfing syndrome was encoded as a categorical variable and SNV‐based metrics as numerical variables. For each species, the mean value of these metrics across RNA‐seq samples was calculated and used as input to the phylopars.lm function. The evolutionary dynamics of these metrics were modelled using a Brownian motion process (see the electronic supplementary material, Dataset for detailed code).

## 3. Results

### (a) Uniform simple sequence repeat genotypes indicate recent transitions to complete cleistogamy

To detect *de novo* mutations over short timescales, we used SSR marker analysis. Despite the high mutation rate of SSR markers, which would typically allow the accumulation of new alleles even in the absence of outcrossing, our analysis of 18 loci revealed that the cleistogamous species *G. takeshimensis* and *G. kuroshimensis* exhibited identical multilocus genotypes across individuals from different populations (electronic supplementary material, table S3–S4). Our estimates indicate that the number of generations over which this identical multilocus genotype can be maintained is very limited. For an effective population size (*N*ₑ) of 500, the probability (*Pᵢ*) that no new mutation becomes fixed falls below 0.05 by the 21st generation; for *N*ₑ = 100, *Pᵢ* drops below 0.05 by the 101st generation; and for *N*ₑ = 50, by the 202nd generation (electronic supplementary material, figure S1). These findings indicate that the extant populations of the cleistogamous species were established very recently.

By contrast, the closely related chasmogamous species *G. foetida* and *G. fontinalis* exhibited some allele variation in SSR markers despite overall low diversity (electronic supplementary material, tables S3 and S4). Phylogenetic analysis based on SSR genotypes revealed that *G. takeshimensis* forms a clade within a well-supported monophyletic group largely consisting of *G. foetida*, whereas *G. kuroshimensis* showed a noticeable genetic distance from *G. fontinalis* (figure 1c). Allele frequency comparisons indicated that all SSR alleles in *G. takeshimensis* were shared with *G. foetida*, while in *G. kuroshimensis*, 15 of the 18 loci were fixed for unique alleles not shared with *G. fontinalis*.

### (b) Multiplexed ISSR genotyping by sequencing analysis confirms very recent divergence of a completely cleistogamous species

Despite the generally low substitution rate of SNPs, genome-wide SNP data provide a robust dataset for estimating historical genomic changes compared with SSR marker analysis. To achieve more accurate estimates of historical genomic shifts, we employed MIG‐seq analysis. MIG‐seq, a genome-wide genotyping technique that targets inter-simple sequence repeat (ISSR) regions, enables efficient SNP detection with relatively low sequencing effort, even in species with large genome sizes [33,34].

A total of 48 684 524 raw reads, averaging 174 497 ± 2026 reads per sample (mean ± s.d.), were obtained from MIG-seq, and 43 122 384 reads (154 561 ± 1793 reads per sample) were used for further analysis. By creating MIG-seq loci using gstacks, the mean effective per-sample coverage was 31.4× ± 13.4× (mean ± s.d.). After reference-based SNP detection and filtering for four species, 5084 SNPs from 279 samples were selected with *R* = 0.1, and 372 unlinked SNPs from 279 samples were selected with *R* = 0.7. Hierarchical SNP detection in sister species revealed 375 SNPs from 94 samples for *G. foetida* and *G. takeshimensis* and 247 SNPs from 185 samples for *G. fontinalis* and *G. kuroshimensis*.

The maximum-likelihood phylogenetic analysis, including morphologically similar *Gastrodia* species, confirmed that *G. kuroshimensis* is most closely related to *G. fontinalis*, whereas *G. takeshimensis* is most closely related to *G. foetida*, though their separation is incomplete (electronic supplementary material, figure S2). This broader analysis supports focusing on these four species to investigate the origins of complete cleistogamy.

**Figure 2.**
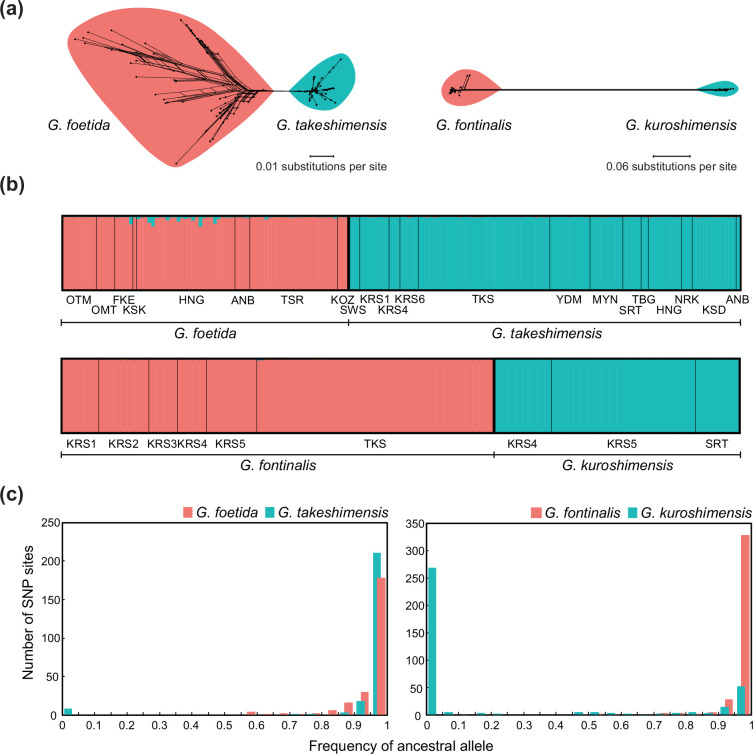
Genomic profiles of the four *Gastrodia* species based on MIG-seq data. (a) Neighbour-net network for sister species pairs (*G. foetida–G. takeshimensis* and *G. fontinalis–G. kuroshimensis*), based on uncorrected *p* distances. Branch lengths represent the average number of substitutions per site. (b) Population structure analysis, with taxonomic divisions marked by thick black vertical lines and population divisions by thin black vertical lines. (c) Allele frequency based on SNP genotypes, if alleles predominant in chasmogamous species represent the ancestral state. Sampling locations, abbreviated in (a) and (b), are shown in figure 1b. See the electronic supplementary material, figures S3 and S4, for the phylogenetic tree and population structure encompassing all four species.

Both phylogenetic and Structure analyses, whether examining these four species alone or with other morphologically similar taxa, indicate minimal divergence between *G. foetida* and *G. takeshimensis* (electronic supplementary material, figures S2–S4). Given this, hierarchical SNP detection within sister species [60] is preferable for achieving higher resolution through lineage-specific SNPs within each of the two highly differentiated lineages, providing more robust results. Consequently, combined evidence from maximum-likelihood and neighbour-net analyses, along with Structure analyses of sister pairs, identified *G. takeshimensis* and *G. kuroshimensis* as distinct phylogenetic groups, clearly separated from their chasmogamous relatives (figure 2a,b; electronic supplementary material, figure S5), with no sign of introgression.

Analysis of population statistics revealed consistently low levels of genetic diversity across all four *Gastrodia* species (electronic supplementary material, figures S5–S8). Notably, the genetic diversity metrics for *G. takeshimensis* were consistently lower than those for *G. foetida*, while *G. kuroshimensis* exhibited higher diversity than *G. fontinalis*. Furthermore, *G. foetida* populations in the southern Ryukyu Islands (OTM and OMT) showed relatively high genetic diversity indices, whereas populations from other regions displayed low diversity similar to that of *G. takeshimensis*. Allele frequency comparisons revealed that most SNP sites in *G. takeshimensis* were dominated by the same alleles as in *G. foetida*, with only four SNPs fixed for opposite alleles, whereas 59% of SNP sites (223 out of 375) in *G. kuroshimensis* were fixed for unique alleles (figure 2c), supporting greater genetic divergence between *G. kuroshimensis* and *G. fontinalis*.

Demographic history analysis using ABC methods estimated the divergence time between *G. foetida* and *G. takeshimensis* to be approximately 35 generations ago, with a 95% credible interval of 15 to 100 generations (electronic supplementary material, table S9). However, the divergence time estimation for *G. kuroshimensis* and *G. fontinalis* was inconclusive because the principal component analysis (PCA) of simulated data did not overlap with the observed data point, indicating model non-convergence (electronic supplementary material, figure S6 and table S10).

### (c) RNA-seq analysis reveals a genomic selfing syndrome in both chasmogamous and completely cleistogamous *Gastrodia* species

**Figure 3.**
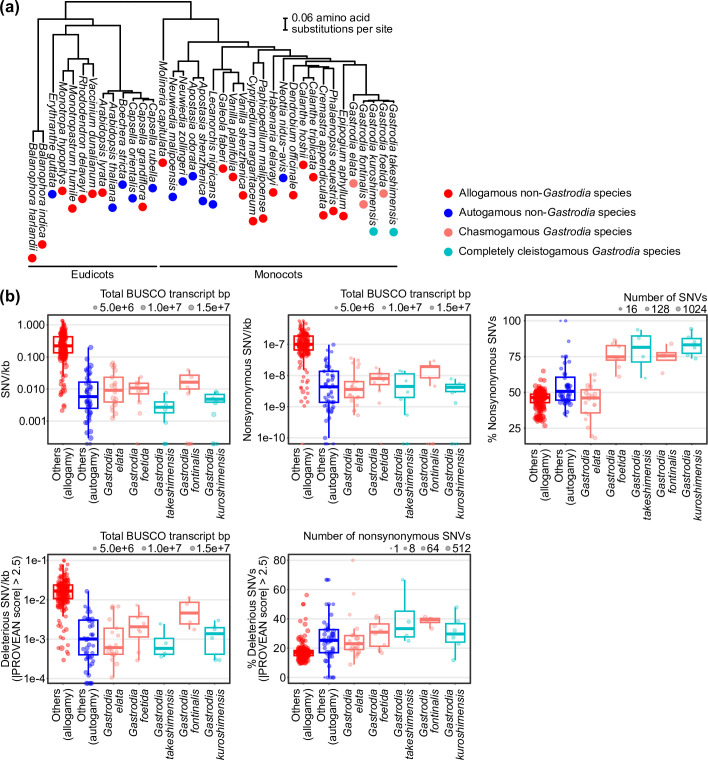
Heterozygous SNV profiles in *Gastrodia* and other angiosperm lineages. (a) Phylogenetic relationships among analysed species. All branches in the maximum-likelihood tree are supported by 100% bootstrap values. (b) Genetic diversity and predicted deleterious variation in *Gastrodia* and other autogamous and allogamous angiosperms, inferred from BUSCO transcripts based on RNA-seq data. Variant frequencies are presented either normalized per kilobase of transcript or as percentages of total SNVs. Each point represents an individual RNA-seq sample. In the box plots, the centre line denotes the median, box boundaries correspond to the upper and lower quartiles, and whiskers indicate 1.5 times the interquartile range.

To define the signature of a genomic selfing syndrome, we analysed heterozygous SNV metrics from publicly available RNA‐seq data for representative self‐pollinating lineages (figure 3a). Because the genomic selfing syndrome is expected to affect both the frequency and fitness effects of SNVs, we focused specifically on SNVs with potentially significant impacts, including non-synonymous and computationally predicted deleterious variants. PGLS analysis revealed significantly lower numbers of SNVs per unit transcript length in self-pollinating species (*p* < 0.001; electronic supplementary material, table S8), along with a higher proportion of non-synonymous SNVs (*p* < 0.001) and deleterious SNVs (*p* < 0.001) across the angiosperms analysed (figure 3b; electronic supplementary material, table S11). Since *de novo* mutations tend to be more deleterious than variants maintained in outcrossing populations [14,15], the higher rates of deleterious mutations in selfing lineages probably result from the rapid generation of homozygous alleles via selfing. Although selfing lineages had significantly lower numbers of deleterious SNVs per kb (*p* < 0.001), this pattern probably reflects an overall reduction in heterozygous variation.

**Figure 4.**
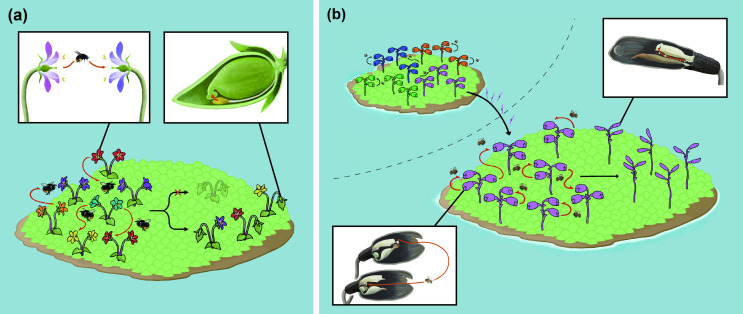
Conceptual diagram illustrating the evolution of complete cleistogamy. (a) General scenario: in most angiosperms, plants producing both cleistogamous and chasmogamous flowers may evolve from ancestrally chasmogamous forms; however, the evolution of complete cleistogamy is probably constrained genetically. (b) *Gastrodia* scenario: drosophilid pollination and reduced genetic diversity owing to founder effects probably promoted the shift to complete cleistogamy. Although chasmogamous *Gastrodia* species lack autonomous selfing, the limited mobility of drosophilid pollinators leads to high geitonogamy, functionally equivalent to self-pollination. These species also exhibit reduced genetic diversity at range margins. Combined with benefits such as reproductive assurance, colonization ability and reinforcement, these factors probably drove the evolution of complete cleistogamy. Flower colours symbolically represent genetic diversity. Designed by Shun Anzai, Kenji Suetsugu and Shingo Kaneko.

Interestingly, the genetic characteristics of *Gastrodia* deviated from the typical distinctions between autogamous and allogamous species. Except for SNVs per kb and the proportion of transcripts with SNVs, where *G. takeshimensis* showed significantly lower values than its chasmogamous sister species (*p* < 0.05 for both metrics), the metrics in all other analysed categories were similar, with no significant differences between the cleistogamous species and their chasmogamous relatives (figure 3; electronic supplementary material, table S12). This suggests that the genomic selfing syndrome may have been established before the emergence of complete cleistogamy (figure 4). Moreover, the signature of the genomic selfing syndrome was consistently detected across species with various nutritional modes, including non-photosynthetic species outside the genus *Gastrodia* (electronic supplementary material, figures S7 and S8), indicating that heterotrophy itself has minimal impact on these genomic profiles.

## 4. Discussion

### (a) Genetic profiles and evolutionary history of completely cleistogamous selfers

We used integrative genomic approaches to elucidate the evolutionary history of *Gastrodia* lineages exhibiting complete cleistogamy. Although these lineages (i.e. *G. takeshimensis* and *G. kuroshimensis*) are distributed across multiple isolated islands, each appears to have independently evolved obligate selfing only once. Notably, these species exhibit no evidence of introgression with their chasmogamous relatives, even in cases of close sympatry [22,23]. In contrast to other selfing lineages that produce some cleistogamous flowers, where introgression may persist [11,19], the strict floral closure in *Gastrodia* probably impedes interspecific gene flow, indicating that complete cleistogamy serves as a robust reproductive barrier. Their distribution patterns further suggest that cleistogamy strengthens species boundaries in regions where closely related chasmogamous lineages coexist.

Our SSR-based analysis suggests that obligate selfing arose very recently in both *G. takeshimensis* and *G. kuroshimensis*. Despite the high mutation rates typically associated with SSR markers, we detected remarkable genetic uniformity among all sampled individuals. Even under conservative estimates involving low mutation rates and small effective population sizes, the likelihood of preserving identical SSR genotypes beyond approximately 200 generations remains exceedingly low. Given a generation time of roughly 5−10 years [45,82], it is probable that *G. takeshimensis* and *G. kuroshimensis* adopted complete cleistogamy within the past 2000 years. Supporting this, MIG‐seq data indicate that *G. takeshimensis* diverged from its chasmogamous sister species only about 35 generations ago, suggesting that the transition to complete cleistogamy initiated speciation. By contrast, *G. kuroshimensis* exhibits pronounced allelic divergence from *G. fontinalis*, indicating that *G. fontinalis* is unlikely to be its direct ancestor; instead, its true sister species may be an unexamined chasmogamous lineage that diverged early from *G. fontinalis* and subsequently became extinct.

While the genomic signatures of *G. takeshimensis* and *G. kuroshimensis* strongly support their obligate selfing nature, the chasmogamous species *G. foetida* and *G. fontinalis* also display genomic profiles indicative of predominant selfing, including reduced genetic diversity and low heterozygosity. Similar patterns were observed in nonsynonymous substitutions and predicted deleterious mutations—features expected to accumulate under sustained self-pollination [3,13]. These trends suggest that self-fertilization or mating among genetically similar individuals is widespread not only in the two cleistogamous species but also in their chasmogamous relatives. Accordingly, the chasmogamous ancestors of the current cleistogamous lineages were probably already predisposed to selfing-like reproductive modes, facilitating the subsequent evolution of complete cleistogamy.

### (b) Driving factors of the evolution of complete cleistogamy

Although *G. foetida* and *G. fontinalis* exhibit genomic profiles typically observed in predominantly selfing plants, they retain floral structures (e.g. a prominent rostellum) that prevent autonomous self-pollination [23]. This seemingly paradoxical combination of ‘outcrossing flowers’ and inbreeding-like genetic features can be attributed to geitonogamy and mating among genetically similar neighbours mediated by *Drosophila* fruit flies, the principal pollinators in these species [40]. The drosophilid pollination system, while adapted to low-light environments, probably results in restricted pollen dispersal and high geitonogamy owing to the limited movement of these flies [40,83]. Given that complete cleistogamy ensures a nearly 100% fruit set, in contrast to the relatively low success of *Drosophila* pollination [22,84], selfing is probably favoured as a form of reproductive assurance [85].

Small island populations represent another likely driver. Island endemics often exhibit reduced genetic diversity owing to founder effects, geographical isolation and small effective population sizes [86]. Interestingly, *G. takeshimensis*, *G. kuroshimensis* and other cleistogamous taxa (e.g. *Gastrodia amamiana*, *Gastrodia clausa* and *Gastrodia flexistyloides*) predominantly inhabit small islands in the Ryukyu Islands [25–29]. Moreover, while chasmogamous *Gastrodia* species (notably *G. foetida*) show moderate genetic diversity on certain islands (e.g. Iriomote Island for *G. foetida* and Takeshima Island for *G. fontinalis*), populations that co-occur with cleistogamous sister species exhibit reduced diversity (e.g. Yakushima Island for *G. foetida* and Kuroshima Island for *G. fontinalis*). The diminished genetic variation in these populations may have rendered mating among different individuals functionally nearly equivalent to self-fertilization, thereby facilitating the transition to complete cleistogamy.

Completely cleistogamous species also appear well-adapted for colonizing new islands, as selfing allows a single individual to establish a population without reliance on mates or pollinators [7,8,87]. Notably, while *G. takeshimensis* and *G. kuroshimensis* often co-occur with chasmogamous sister species, they are also distributed on islands lacking such relatives (e.g. *G. takeshimensis* on Takeshima, Kuroshima and Suwanosejima Islands and *G. kuroshimensis* on Yakushima Island) [26,28,42,43].

Finally, autonomous selfing mitigates reproductive interference, such as heterospecific pollen deposition that can clog stigmas, inhibit conspecific pollen germination or usurp ovules, thereby facilitating the coexistence of closely related species in some cases [88,89]. Among selfing strategies, complete cleistogamy is particularly effective at precluding heterospecific pollen receipt [22]. Given that the divergence between the ancestors of *G. fontinalis* and *G. kuroshimensis* probably predates the origin of complete cleistogamy in *G. kuroshimensis*, it is plausible that the lineages initially diverged allopatrically for reasons unrelated to cleistogamy. Subsequent secondary contact may have selected for complete cleistogamy in the direct ancestor of *G. kuroshimensis* (now probably extinct) as a reinforcement mechanism.

Taken together, the recurrent emergence of completely cleistogamous taxa in *Gastrodia* probably reflects a trade-off between immediate and long-term fitness benefits [22]. Once a lineage becomes genetically uniform and pollinator-mediated outcrossing is inherently restricted, the evolution of complete cleistogamy may confer advantages such as reproductive assurance, enhanced colonization ability and strong reproductive isolation in multispecies communities. However, the extremely recent origin (<200 generations) of complete cleistogamy in *G. takeshimensis* and *G. kuroshimensis* suggests that obligate selfing may be evolutionarily ephemeral. As even minimal levels of outcrossing can reduce the accumulation of deleterious mutations [14,15], complete cleistogamy might entail long-term genetic vulnerabilities.

Considering the rarity of complete cleistogamy and the historical scepticism about its existence since Darwin [9], comparative studies, including analyses of other lineages (e.g. *Gastrodia indica*) [90], will be essential to clarify the ecological and genetic conditions that promote its evolution (e.g. whether reduced genetic diversity precedes its emergence) and to determine the timescales over which such lineages can persist without outcrossing. Moreover, while this study sheds light on the ultimate (evolutionary) drivers of complete cleistogamy, complementary approaches such as interspecific crossing and quantitative trait locus (QTL) mapping [91] will be instrumental in uncovering the proximate mechanisms and genomic architecture underlying this reproductive strategy.

## Data Availability

The raw sequencing data have been deposited in the DDBJ Sequence Read Archive under BioProject accession numbers PRJDB18852 for MIG-seq, and PRJDB10965, PRJDB10966, PRJDB10992 and PRJDB10993 for RNA-seq. Additional data from this study are available on Figshare [92]. Supplementary material is available online [93].
